# Beware of missed diagnosis in patients with multiple genetic diseases: a case report

**DOI:** 10.1186/s12887-022-03490-0

**Published:** 2022-07-20

**Authors:** Detong Guo, Xuemei Li, Nan Liu, Xiaoli Yu, Jianbo Shu, Wenchao Sheng, Dong Li, Chunquan Cai

**Affiliations:** 1 Tianjin Children’s Hospital (Children’s Hospital of Tianjin University), Beichen District, No. 238 Longyan Road, Tianjin, 300134 China; 2 Graduate College of Tianjin Medical University, Tianjin, 300070 China; 3 Department of Neurology, Tianjin Children’s Hospital, Beichen District, No. 238 Longyan Road, Tianjin, 300134 China; 4 Tianjin Pediatric Research Institute, Tianjin, 300134 China; 5 Tianjin Key Laboratory of Birth Defects for Prevention and Treatment, Tianjin, 300134 China; 6 Department of Neurosurgery, Tianjin Children’s Hospital, Tianjin, 300134 China

**Keywords:** Duchenne muscular dystrophies, Cerebral cavernous malformations, *DMD* gene, *PDCD*10 gene, Whole-exome sequencing, Case report

## Abstract

**Background:**

Duchenne muscular dystrophy (DMD) is an X-linked recessive inherited disorder caused by the absence of the Dystrophin protein. Cerebral cavernous malformations (CCMs) are the most common vascular abnormalities in the central nervous system caused by the absence of the products of the *CCM* genes. Most CCMs cases reported occurring in a sporadic form are often asymptomatic.

**Case presentation:**

We report a rare case of a 7-year-old Chinese boy with a co-existing DMD and sporadic CCMs. We found classic clinical features of DMD and non-specific pathological changes in his brain. We made the definitive diagnosis based on the results of whole-exome sequencing (WES), a repeat from exon 3 to exon 9 of the *DMD* inherited from his mother, and a de novo heterozygote nonsense mutation C.418G > T of the *PDCD10* exon 6.

**Conclusion:**

We should take care to avoid missed diagnoses in patients with multiple genetic disorders.

## Background

Duchenne muscular dystrophy (DMD) is an X-linked recessive inherited disorder caused by variants in *DMD*. *DMD* is the longest human gene on the locus p21 of chromosome X. It comprises 79 exons and encodes the dystrophin protein [1]. DMD is a devastating disease characterized by serum creatine kinase elevation (50 to 100 times the average values), altered gait, calf enlargement (commonly called “pseudohypertrophy”), tight heel cords, and lordosis [2]. The newborn screening studies show that the incidence of DMD ranges from 1:3500 to 1:5000 among live male births [3]. DMD can be diagnosed by observed clinical features, increased serum creatine kinase (CK) levels, muscle biopsy, and genetic tests [4].

Cerebral cavernous malformations (CCMs) are the most common vascular abnormalities [5]. We can find patients with familial forms of CCMs with evident clinical symptoms, such as seizures, intracerebral hemorrhage(ICH), and focal neurological deficits(FND) [6]. However, about 80% of CCMs patients were reported in sporadic forms and had no symptoms. Clinical symptoms of sporadic CCMs can present at any age, typically appearing between 20 and 30 years of age [7]. According to recent studies, variants in 3 genes, *CCM1/KRIT1*, *CCM2*, and *CCM3/PDCD10*, were found in 30–40% of sporadic CCMs patients [8], the products of *CCM3/PDCD10* have been proven to play an essential role in regulating angiogenesis [9] and stress response [10]. So variants in the *PDCD10* gene are thought to be closely related to CCMs.

Although the pathogenesis of CCMs is unclear and may be related to many factors, it is acknowledged that mainly related to the variations of *CCM* genes. The variant in any *CCM* genes may lead to CCMs because all these genes are involved in angiogenesis. ClinVar database has recorded 254 single gene variants of *CCM* genes at present, 113 of which are pathogenic. Exonic variants have been reported to be responsible for most monogenic diseases. About 60% of the monogenic diseases are caused by exome’s missense and nonsense variants [11]. With the development of whole-exome sequencing (WES), it has been more frequently used in clinical diagnostics, especially applicable to patients who have atypical presentations of a genetic disorder or the early diagnostic evaluation of a disease when the classic manifestation has not appeared [12]. Given the late average age of onset of sporadic CCMs and the absence of apparent symptoms prior to onset, WES can be used as an essential means of early screening for sporadic CCMs, especially for those patients with cerebral vascular pathological changes but have no intracranial hemorrhage or other clinical symptoms, and without CCMs positive family history.

Here, we report a patient who has two diseases simultaneously, hereditary DMD and sporadic CCMs. The patient had typical clinical manifestations of DMD but was short of apparent symptoms of CCMs. The final diagnosis of sporadic CCMs was made by combining the detection results of WES.

## Case presentation

A 7-year-old Chinese boy was born to healthy non-consanguineous parents. The patient developed weakness in both lower limbs at the age of 2. When he was 6, the patient showed typical Gowers’ sign (difficulties going upstairs, gait with peculiar oscillating characters, and characteristic ways to rise from the floor). With the deterioration of the disease, the patient cannot run or jump at present, and reduced strength in both upper limbs renders the patient unable to hold objects stably. The boy was born in good condition, and his family history was not significant.

**Fig.1:**
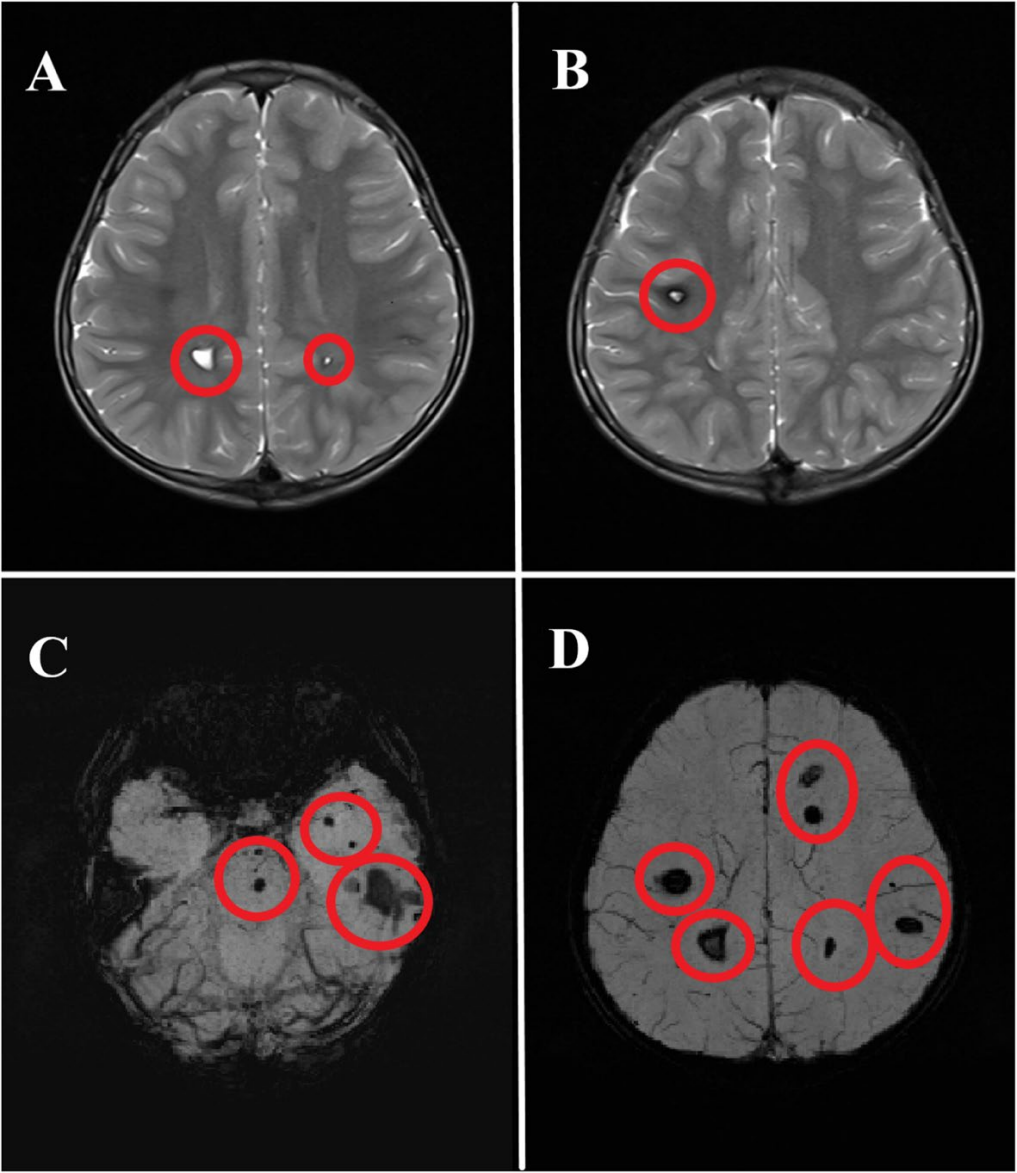
The abnormal magnetic resonance imaging (MRI) of head. **a** T2WI showed high signal intensity in bilateral frontal-parietal lobes (marked with red circles). **b** T2WI showed high signal intensity in the right frontal-parietal lobe (marked with a red circle). **c** SWI showed low signal intensity in multiple sites of the brain stem and bilateral temporal lobes (marked with red circles). **d** SWI showed low signal intensity in multiple sites of bilateral frontal-parietal lobes (marked with red circles)

The patient showed bilateral gastrocnemius muscle hypertrophy, contracture of the bilateral ankle joint, and foot drop. In addition, the patient had a significantly increased serum CK (creatine kinase) value, about 34 times the average level. We preliminarily diagnosed the patient with DMD according to the characteristic signs we observed. We performed electromyography and magnetic resonance imaging on the patient. The results also showed pathological changes of bilateral lower limbs that supported our diagnosis. Unexpectedly, we also found multiple abnormal signals in the patient’s frontotemporal and parietal lobes, brainstem, right basal ganglia, and right cerebellar hemisphere (Fig. 1).

**Fig.2:**
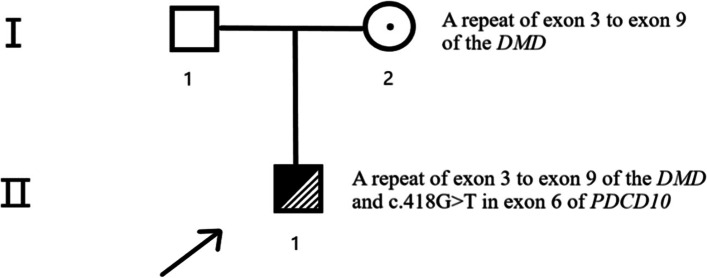
Pedigree chart. The father (I1) was healthy (marked as a hollow square). The mother (I2) was healthy (marked as a black dot in a circle) even though she carried a repeat of exon 3 to exon 9 of the *DMD*. The patient (II1, pointed out by the arrow) inherited the variant from his mother and carried another de novo variant, a heterozygous nonsense mutation c.418G > T in exon 6 of *PDCD10*, and the patient developed DMD and CCMs (marked as half dark and half shadow)

To further clarify the diagnosis, we performed a WES examination on the patient and their parents after obtaining the consent of their families. The results showed that the patient had a repeat of exon 3 to exon 9 of the *DMD* from the patient’s mother, consistent with our previous diagnosis. Interestingly, we also found another variant, a heterozygous nonsense mutation c.418G > T in exon 6 of *PDCD10*, absent in the patient’s parents. (The pedigree chart in this family is shown in Fig. 2). Since the variants of *PDCD10* are related to CCMs, combined with the vascular abnormalities found in the patient’s head, we clarified that this patient is with both DMD and CCMs.

Because the patient did not show the symptoms of CCMs, we adopted a conservative strategy and recommended the patient review regularly to observe progression. As for DMD, it is still incurable. We had symptomatic treatment for the patient, but the muscle weakness shown by the patient was not relieved. The patient’s family refused further treatment for personal reasons and was automatic discharge. A follow-up visit was not available.

It is worth noting that the patient is 115 cm ( -3SD) in height and 18.1 kg (-3SD) in weight, with prominent short stature and retarded bone maturation. We performed some endocrine tests, such as thyroid function and growth hormone levels. However, the results could not explain these abnormalities.

## Discussion and conclusion

DMD is the most common form of muscular dystrophies among children [13] When they were born, boys with DMD have normal muscle function but a progressive adiposification and hypofunction of muscle tissue [14]. We can diagnose DMD through typical clinical symptoms while sporadic CCMs often absent clinical manifestations before onset. Our patient was such a very rare case who had these two diseases simultaneously.

Our patient’s clinical manifestations were highly consistent with the characteristic clinical phenotypes of DMD so we can get a precise diagnosis quickly. At the same time, however, it is easy to overlook the possibility that the patient may also suffer from other genetic diseases, if we did not do WES for further definite diagnosis of DMD for the patient, the diagnosis of sporadic CCMs would have been missed. It has been reported that many patients have over one pathogenic variant, but they did not get the correct test results initially. [15]. Therefore, as WES has been proven to discover new mutations and pathways in many disease domains [16], we need to take advantage of WES’s ability to find multiple mutation sites. Thus, we can reduce the possibility of missed diagnosis for patients who have two or several genetic diseases but lack some evident symptoms. Timely and accurate genetic diagnosis often affects the prognosis of patients [17].

As for the patient’s short stature with an unknown mechanism, our study found that though short stature is not a recognized clinical phenotypic feature of DMD, it is commonly found in DMD patients [18–20], so we have reason to suspect that the patient’s short stature may be related to DMD. However, it is essential to note that the short stature of our patient had something different from previous studies, especially our patient had significant bone age delays, while the reported DMD patients with short stature were characterized by normal bone maturation [19]. Besides, in previous reports, the height of DMD patients remains stable at about -1.5SD after 3 or 4 years of age. In contrast, our patient reached -3 SD, which was much more severe [19], these differences lead us to consider whether endocrine abnormalities play a role in our patient’s short stature. However, the normal results of growth hormone stimulation tests and the absence of genetic variants associated with short stature in the patient led us to rule out the possibility of endocrine-related short stature in the patient [21, 22]. Unfortunately, the pathogenesis of short stature in DMD patients and its potential impact on the disease process have not been well understood yet, so we have no way to explain it with further validation [18].

In general, although there are few cases similar to our reported case, we should still be on guard against the missed diagnoses of such patients. When we find nonspecific pathological changes in patients who have no symptoms, WES may help make a precise diagnosis after excluding other possible diseases. Furthermore, WES can also be used for early diagnosis and risk assessment for diseases like sporadic CCMs with late-onset and lack of obvious clinical symptoms before onset. Appropriate intervention for high-risk patients before bleeding may improve the prognosis of patients.

We should pay attention to avoid misdiagnosis in patients with multiple genetic diseases, especially some patients who lack typical clinical manifestations. Appropriate WES can help us to diagnose and guide therapy.

## Data Availability

The datasets generated and/or analysed during the current study are available in the ClinVar repository, SCV002538922 and SCV002538923.
